# Supplementary material for: Pyruvate Dehydrogenase Kinase 1 inhibition mediated oxidative phosphorylation enhancement in cartilage promotes osteoarthritis progression
Source: BMC Musculoskelet Disord. 2023 Jul 20;24:597. doi: 10.1186/s12891-023-06585-6 (PMC10357736; doi:10.1186/s12891-023-06585-6)
Supplement: Supplementary file 1 — Additional file 1. [file 12891_2023_6585_MOESM1_ESM.zip › BP.Gsea.1653623667859/GOBP_MITOCHONDRION_ORGANIZATION.html]

Details for gene set GOBP\_MITOCHONDRION\_ORGANIZATION[GSEA]

|  || Dataset | 55457\_collapsed\_to\_symbols.cls.cls #OA\_versus\_normal.cls.cls #OA\_versus\_normal\_repos |
| Phenotype | cls.cls#OA\_versus\_normal\_repos |
| Upregulated in class | OA |
| GeneSet | GOBP\_MITOCHONDRION\_ORGANIZATION |
| Enrichment Score (ES) | 0.28404462 |
| Normalized Enrichment Score (NES) | 1.591363 |
| Nominal p-value | 0.0 |
| FDR q-value | 0.27218944 |
| FWER p-Value | 1.0 |
Table: GSEA Results Summary

  

Fig 1: Enrichment plot: GOBP\_MITOCHONDRION\_ORGANIZATION      
 Profile of the Running ES Score & Positions of GeneSet Members on the Rank Ordered List

  

| SYMBOL | TITLE | RANK IN GENE LIST | RANK METRIC SCORE | RUNNING ES | CORE ENRICHMENT || 1 | FAM162A | family with sequence similarity 162 member A [Source:HGNC Symbol;Acc:HGNC:17865] | 4 | 1.258 | 0.0096 | Yes |
| 2 | PARL | presenilin associated rhomboid like [Source:HGNC Symbol;Acc:HGNC:18253] | 14 | 1.156 | 0.0180 | Yes |
| 3 | PSMD10 | "proteasome 26S subunit, non-ATPase 10 [Source:HGNC Symbol;Acc:HGNC:9555]" | 58 | 0.976 | 0.0223 | Yes |
| 4 | COX17 | cytochrome c oxidase copper chaperone COX17 [Source:HGNC Symbol;Acc:HGNC:2264] | 64 | 0.966 | 0.0295 | Yes |
| 5 | NDUFA8 | NADH:ubiquinone oxidoreductase subunit A8 [Source:HGNC Symbol;Acc:HGNC:7692] | 67 | 0.961 | 0.0369 | Yes |
| 6 | SAMM50 | SAMM50 sorting and assembly machinery component [Source:HGNC Symbol;Acc:HGNC:24276] | 99 | 0.915 | 0.0417 | Yes |
| 7 | MTX1 | metaxin 1 [Source:HGNC Symbol;Acc:HGNC:7504] | 116 | 0.892 | 0.0474 | Yes |
| 8 | TMEM186 | transmembrane protein 186 [Source:HGNC Symbol;Acc:HGNC:24530] | 128 | 0.876 | 0.0534 | Yes |
| 9 | TMEM14A | transmembrane protein 14A [Source:HGNC Symbol;Acc:HGNC:21076] | 161 | 0.848 | 0.0576 | Yes |
| 10 | NDUFS3 | NADH:ubiquinone oxidoreductase core subunit S3 [Source:HGNC Symbol;Acc:HGNC:7710] | 177 | 0.836 | 0.0630 | Yes |
| 11 | MPV17 | mitochondrial inner membrane protein MPV17 [Source:HGNC Symbol;Acc:HGNC:7224] | 182 | 0.830 | 0.0692 | Yes |
| 12 | NDUFA1 | NADH:ubiquinone oxidoreductase subunit A1 [Source:HGNC Symbol;Acc:HGNC:7683] | 194 | 0.826 | 0.0748 | Yes |
| 13 | GHITM | growth hormone inducible transmembrane protein [Source:HGNC Symbol;Acc:HGNC:17281] | 213 | 0.809 | 0.0798 | Yes |
| 14 | RALBP1 | ralA binding protein 1 [Source:HGNC Symbol;Acc:HGNC:9841] | 217 | 0.808 | 0.0859 | Yes |
| 15 | EYA2 | EYA transcriptional coactivator and phosphatase 2 [Source:HGNC Symbol;Acc:HGNC:3520] | 219 | 0.807 | 0.0922 | Yes |
| 16 | PANK2 | pantothenate kinase 2 [Source:HGNC Symbol;Acc:HGNC:15894] | 220 | 0.805 | 0.0986 | Yes |
| 17 | NDUFA13 | NADH:ubiquinone oxidoreductase subunit A13 [Source:HGNC Symbol;Acc:HGNC:17194] | 236 | 0.795 | 0.1036 | Yes |
| 18 | NDUFA10 | NADH:ubiquinone oxidoreductase subunit A10 [Source:HGNC Symbol;Acc:HGNC:7684] | 243 | 0.788 | 0.1094 | Yes |
| 19 | FIS1 | "fission, mitochondrial 1 [Source:HGNC Symbol;Acc:HGNC:21689]" | 250 | 0.785 | 0.1151 | Yes |
| 20 | NDUFB6 | NADH:ubiquinone oxidoreductase subunit B6 [Source:HGNC Symbol;Acc:HGNC:7701] | 262 | 0.779 | 0.1203 | Yes |
| 21 | NDUFAF3 | NADH:ubiquinone oxidoreductase complex assembly factor 3 [Source:HGNC Symbol;Acc:HGNC:29918] | 285 | 0.761 | 0.1246 | Yes |
| 22 | TMEM126B | transmembrane protein 126B [Source:HGNC Symbol;Acc:HGNC:30883] | 295 | 0.753 | 0.1298 | Yes |
| 23 | TREM2 | triggering receptor expressed on myeloid cells 2 [Source:HGNC Symbol;Acc:HGNC:17761] | 342 | 0.726 | 0.1319 | Yes |
| 24 | HAX1 | HCLS1 associated protein X-1 [Source:HGNC Symbol;Acc:HGNC:16915] | 387 | 0.711 | 0.1340 | Yes |
| 25 | SLC25A31 | solute carrier family 25 member 31 [Source:HGNC Symbol;Acc:HGNC:25319] | 395 | 0.708 | 0.1390 | Yes |
| 26 | COA1 | cytochrome c oxidase assembly factor 1 [Source:HGNC Symbol;Acc:HGNC:21868] | 426 | 0.695 | 0.1421 | Yes |
| 27 | NDUFB3 | NADH:ubiquinone oxidoreductase subunit B3 [Source:HGNC Symbol;Acc:HGNC:7698] | 483 | 0.667 | 0.1428 | Yes |
| 28 | COX16 | cytochrome c oxidase assembly factor COX16 [Source:HGNC Symbol;Acc:HGNC:20213] | 498 | 0.660 | 0.1469 | Yes |
| 29 | MTX2 | metaxin 2 [Source:HGNC Symbol;Acc:HGNC:7506] | 518 | 0.654 | 0.1506 | Yes |
| 30 | NDUFS4 | NADH:ubiquinone oxidoreductase subunit S4 [Source:HGNC Symbol;Acc:HGNC:7711] | 531 | 0.647 | 0.1547 | Yes |
| 31 | NDUFA3 | NADH:ubiquinone oxidoreductase subunit A3 [Source:HGNC Symbol;Acc:HGNC:7686] | 550 | 0.641 | 0.1583 | Yes |
| 32 | COA3 | cytochrome c oxidase assembly factor 3 [Source:HGNC Symbol;Acc:HGNC:24990] | 597 | 0.625 | 0.1596 | Yes |
| 33 | HIGD2A | HIG1 hypoxia inducible domain family member 2A [Source:HGNC Symbol;Acc:HGNC:28311] | 614 | 0.618 | 0.1632 | Yes |
| 34 | APOO | apolipoprotein O [Source:HGNC Symbol;Acc:HGNC:28727] | 646 | 0.606 | 0.1655 | Yes |
| 35 | TSPO | translocator protein [Source:HGNC Symbol;Acc:HGNC:1158] | 692 | 0.595 | 0.1666 | Yes |
| 36 | LRBA | LPS responsive beige-like anchor protein [Source:HGNC Symbol;Acc:HGNC:1742] | 701 | 0.594 | 0.1706 | Yes |
| 37 | GABARAPL2 | GABA type A receptor associated protein like 2 [Source:HGNC Symbol;Acc:HGNC:13291] | 776 | 0.575 | 0.1693 | Yes |
| 38 | RAB32 | "RAB32, member RAS oncogene family [Source:HGNC Symbol;Acc:HGNC:9772]" | 817 | 0.564 | 0.1705 | Yes |
| 39 | ZDHHC6 | zinc finger DHHC-type palmitoyltransferase 6 [Source:HGNC Symbol;Acc:HGNC:19160] | 837 | 0.559 | 0.1734 | Yes |
| 40 | NDUFA2 | NADH:ubiquinone oxidoreductase subunit A2 [Source:HGNC Symbol;Acc:HGNC:7685] | 878 | 0.549 | 0.1745 | Yes |
| 41 | NDUFB5 | NADH:ubiquinone oxidoreductase subunit B5 [Source:HGNC Symbol;Acc:HGNC:7700] | 902 | 0.542 | 0.1770 | Yes |
| 42 | CAMK2A | calcium/calmodulin dependent protein kinase II alpha [Source:HGNC Symbol;Acc:HGNC:1460] | 916 | 0.538 | 0.1802 | Yes |
| 43 | TACO1 | translational activator of cytochrome c oxidase I [Source:HGNC Symbol;Acc:HGNC:24316] | 923 | 0.536 | 0.1839 | Yes |
| 44 | NDUFC2 | NADH:ubiquinone oxidoreductase subunit C2 [Source:HGNC Symbol;Acc:HGNC:7706] | 929 | 0.532 | 0.1877 | Yes |
| 45 | MFF | mitochondrial fission factor [Source:HGNC Symbol;Acc:HGNC:24858] | 932 | 0.531 | 0.1918 | Yes |
| 46 | NDUFA9 | NADH:ubiquinone oxidoreductase subunit A9 [Source:HGNC Symbol;Acc:HGNC:7693] | 943 | 0.529 | 0.1951 | Yes |
| 47 | DNAJC15 | DnaJ heat shock protein family (Hsp40) member C15 [Source:HGNC Symbol;Acc:HGNC:20325] | 954 | 0.527 | 0.1985 | Yes |
| 48 | NDUFB4 | NADH:ubiquinone oxidoreductase subunit B4 [Source:HGNC Symbol;Acc:HGNC:7699] | 968 | 0.525 | 0.2016 | Yes |
| 49 | ARMC1 | armadillo repeat containing 1 [Source:HGNC Symbol;Acc:HGNC:17684] | 1004 | 0.517 | 0.2029 | Yes |
| 50 | NDUFB7 | NADH:ubiquinone oxidoreductase subunit B7 [Source:HGNC Symbol;Acc:HGNC:7702] | 1033 | 0.510 | 0.2047 | Yes |
| 51 | CALM3 | calmodulin 3 [Source:HGNC Symbol;Acc:HGNC:1449] | 1039 | 0.510 | 0.2083 | Yes |
| 52 | NDUFB1 | NADH:ubiquinone oxidoreductase subunit B1 [Source:HGNC Symbol;Acc:HGNC:7695] | 1055 | 0.506 | 0.2111 | Yes |
| 53 | MAN2A1 | mannosidase alpha class 2A member 1 [Source:HGNC Symbol;Acc:HGNC:6824] | 1069 | 0.501 | 0.2140 | Yes |
| 54 | NDUFS7 | NADH:ubiquinone oxidoreductase core subunit S7 [Source:HGNC Symbol;Acc:HGNC:7714] | 1071 | 0.501 | 0.2179 | Yes |
| 55 | NDUFC1 | NADH:ubiquinone oxidoreductase subunit C1 [Source:HGNC Symbol;Acc:HGNC:7705] | 1091 | 0.499 | 0.2203 | Yes |
| 56 | HSPA1L | heat shock protein family A (Hsp70) member 1 like [Source:HGNC Symbol;Acc:HGNC:5234] | 1139 | 0.488 | 0.2204 | Yes |
| 57 | ATG5 | autophagy related 5 [Source:HGNC Symbol;Acc:HGNC:589] | 1142 | 0.488 | 0.2241 | Yes |
| 58 | UBL5 | ubiquitin like 5 [Source:HGNC Symbol;Acc:HGNC:13736] | 1160 | 0.485 | 0.2265 | Yes |
| 59 | PYCARD | PYD and CARD domain containing [Source:HGNC Symbol;Acc:HGNC:16608] | 1164 | 0.484 | 0.2301 | Yes |
| 60 | AKT1 | AKT serine/threonine kinase 1 [Source:HGNC Symbol;Acc:HGNC:391] | 1192 | 0.478 | 0.2317 | Yes |
| 61 | NDUFS8 | NADH:ubiquinone oxidoreductase core subunit S8 [Source:HGNC Symbol;Acc:HGNC:7715] | 1208 | 0.474 | 0.2343 | Yes |
| 62 | MOAP1 | modulator of apoptosis 1 [Source:HGNC Symbol;Acc:HGNC:16658] | 1215 | 0.473 | 0.2375 | Yes |
| 63 | SH3GLB1 | "SH3 domain containing GRB2 like, endophilin B1 [Source:HGNC Symbol;Acc:HGNC:10833]" | 1216 | 0.473 | 0.2412 | Yes |
| 64 | NDUFS1 | NADH:ubiquinone oxidoreductase core subunit S1 [Source:HGNC Symbol;Acc:HGNC:7707] | 1229 | 0.471 | 0.2440 | Yes |
| 65 | TNFSF10 | TNF superfamily member 10 [Source:HGNC Symbol;Acc:HGNC:11925] | 1241 | 0.468 | 0.2468 | Yes |
| 66 | WDR45 | WD repeat domain 45 [Source:HGNC Symbol;Acc:HGNC:28912] | 1332 | 0.453 | 0.2432 | Yes |
| 67 | PARK7 | Parkinsonism associated deglycase [Source:HGNC Symbol;Acc:HGNC:16369] | 1406 | 0.442 | 0.2408 | Yes |
| 68 | MIEF1 | mitochondrial elongation factor 1 [Source:HGNC Symbol;Acc:HGNC:25979] | 1531 | 0.423 | 0.2343 | Yes |
| 69 | RCC1L | RCC1 like [Source:HGNC Symbol;Acc:HGNC:14948] | 1559 | 0.419 | 0.2354 | Yes |
| 70 | ATP5IF1 | ATP synthase inhibitory factor subunit 1 [Source:HGNC Symbol;Acc:HGNC:871] | 1575 | 0.417 | 0.2375 | Yes |
| 71 | BCAP31 | B cell receptor associated protein 31 [Source:HGNC Symbol;Acc:HGNC:16695] | 1584 | 0.416 | 0.2402 | Yes |
| 72 | NDUFB11 | NADH:ubiquinone oxidoreductase subunit B11 [Source:HGNC Symbol;Acc:HGNC:20372] | 1603 | 0.413 | 0.2420 | Yes |
| 73 | TMEM242 | transmembrane protein 242 [Source:HGNC Symbol;Acc:HGNC:17206] | 1606 | 0.413 | 0.2451 | Yes |
| 74 | MUL1 | mitochondrial E3 ubiquitin protein ligase 1 [Source:HGNC Symbol;Acc:HGNC:25762] | 1610 | 0.412 | 0.2481 | Yes |
| 75 | WIPI2 | "WD repeat domain, phosphoinositide interacting 2 [Source:HGNC Symbol;Acc:HGNC:32225]" | 1612 | 0.411 | 0.2512 | Yes |
| 76 | AIP | aryl hydrocarbon receptor interacting protein [Source:HGNC Symbol;Acc:HGNC:358] | 1649 | 0.406 | 0.2516 | Yes |
| 77 | HAP1 | huntingtin associated protein 1 [Source:HGNC Symbol;Acc:HGNC:4812] | 1658 | 0.405 | 0.2541 | Yes |
| 78 | GPER1 | G protein-coupled estrogen receptor 1 [Source:HGNC Symbol;Acc:HGNC:4485] | 1677 | 0.402 | 0.2559 | Yes |
| 79 | HMGCL | 3-hydroxy-3-methylglutaryl-CoA lyase [Source:HGNC Symbol;Acc:HGNC:5005] | 1703 | 0.398 | 0.2570 | Yes |
| 80 | CXADR | CXADR Ig-like cell adhesion molecule [Source:HGNC Symbol;Acc:HGNC:2559] | 1719 | 0.395 | 0.2589 | Yes |
| 81 | CEBPA | CCAAT enhancer binding protein alpha [Source:HGNC Symbol;Acc:HGNC:1833] | 1750 | 0.392 | 0.2596 | Yes |
| 82 | SIRT5 | sirtuin 5 [Source:HGNC Symbol;Acc:HGNC:14933] | 1758 | 0.390 | 0.2621 | Yes |
| 83 | SNCA | synuclein alpha [Source:HGNC Symbol;Acc:HGNC:11138] | 1800 | 0.384 | 0.2619 | Yes |
| 84 | STOML2 | stomatin like 2 [Source:HGNC Symbol;Acc:HGNC:14559] | 1819 | 0.382 | 0.2635 | Yes |
| 85 | SURF1 | SURF1 cytochrome c oxidase assembly factor [Source:HGNC Symbol;Acc:HGNC:11474] | 1829 | 0.381 | 0.2657 | Yes |
| 86 | AIFM1 | apoptosis inducing factor mitochondria associated 1 [Source:HGNC Symbol;Acc:HGNC:8768] | 1838 | 0.380 | 0.2681 | Yes |
| 87 | TIMM8B | translocase of inner mitochondrial membrane 8 homolog B [Source:HGNC Symbol;Acc:HGNC:11818] | 1877 | 0.376 | 0.2680 | Yes |
| 88 | AP3B1 | adaptor related protein complex 3 subunit beta 1 [Source:HGNC Symbol;Acc:HGNC:566] | 1883 | 0.375 | 0.2706 | Yes |
| 89 | NIPSNAP2 | nipsnap homolog 2 [Source:HGNC Symbol;Acc:HGNC:4179] | 1886 | 0.374 | 0.2734 | Yes |
| 90 | PRKN | parkin RBR E3 ubiquitin protein ligase [Source:HGNC Symbol;Acc:HGNC:8607] | 1972 | 0.364 | 0.2695 | Yes |
| 91 | VPS35 | VPS35 retromer complex component [Source:HGNC Symbol;Acc:HGNC:13487] | 2008 | 0.360 | 0.2695 | Yes |
| 92 | COX7A1 | cytochrome c oxidase subunit 7A1 [Source:HGNC Symbol;Acc:HGNC:2287] | 2045 | 0.356 | 0.2694 | Yes |
| 93 | CLU | clusterin [Source:HGNC Symbol;Acc:HGNC:2095] | 2083 | 0.352 | 0.2692 | Yes |
| 94 | PINK1 | PTEN induced kinase 1 [Source:HGNC Symbol;Acc:HGNC:14581] | 2097 | 0.350 | 0.2710 | Yes |
| 95 | MARCHF5 | membrane associated ring-CH-type finger 5 [Source:HGNC Symbol;Acc:HGNC:26025] | 2150 | 0.344 | 0.2695 | Yes |
| 96 | PRDX3 | peroxiredoxin 3 [Source:HGNC Symbol;Acc:HGNC:9354] | 2161 | 0.343 | 0.2714 | Yes |
| 97 | TRIAP1 | TP53 regulated inhibitor of apoptosis 1 [Source:HGNC Symbol;Acc:HGNC:26937] | 2163 | 0.342 | 0.2741 | Yes |
| 98 | TIMM10 | translocase of inner mitochondrial membrane 10 [Source:HGNC Symbol;Acc:HGNC:11814] | 2244 | 0.333 | 0.2703 | Yes |
| 99 | NDUFS5 | NADH:ubiquinone oxidoreductase subunit S5 [Source:HGNC Symbol;Acc:HGNC:7712] | 2267 | 0.330 | 0.2711 | Yes |
| 100 | CHCHD3 | coiled-coil-helix-coiled-coil-helix domain containing 3 [Source:HGNC Symbol;Acc:HGNC:21906] | 2279 | 0.329 | 0.2728 | Yes |
| 101 | IGF1 | insulin like growth factor 1 [Source:HGNC Symbol;Acc:HGNC:5464] | 2286 | 0.328 | 0.2750 | Yes |
| 102 | FBXO7 | F-box protein 7 [Source:HGNC Symbol;Acc:HGNC:13586] | 2327 | 0.323 | 0.2743 | Yes |
| 103 | HTRA2 | HtrA serine peptidase 2 [Source:HGNC Symbol;Acc:HGNC:14348] | 2350 | 0.320 | 0.2751 | Yes |
| 104 | VPS11 | VPS11 core subunit of CORVET and HOPS complexes [Source:HGNC Symbol;Acc:HGNC:14583] | 2390 | 0.315 | 0.2744 | Yes |
| 105 | TTC19 | tetratricopeptide repeat domain 19 [Source:HGNC Symbol;Acc:HGNC:26006] | 2394 | 0.315 | 0.2767 | Yes |
| 106 | SDHAF3 | succinate dehydrogenase complex assembly factor 3 [Source:HGNC Symbol;Acc:HGNC:21752] | 2409 | 0.313 | 0.2780 | Yes |
| 107 | CHCHD2 | coiled-coil-helix-coiled-coil-helix domain containing 2 [Source:HGNC Symbol;Acc:HGNC:21645] | 2431 | 0.310 | 0.2788 | Yes |
| 108 | NDUFB2 | NADH:ubiquinone oxidoreductase subunit B2 [Source:HGNC Symbol;Acc:HGNC:7697] | 2460 | 0.307 | 0.2790 | Yes |
| 109 | FASTKD3 | FAST kinase domains 3 [Source:HGNC Symbol;Acc:HGNC:28758] | 2473 | 0.306 | 0.2804 | Yes |
| 110 | CAMKK2 | calcium/calmodulin dependent protein kinase kinase 2 [Source:HGNC Symbol;Acc:HGNC:1470] | 2497 | 0.303 | 0.2810 | Yes |
| 111 | COX7A2L | cytochrome c oxidase subunit 7A2 like [Source:HGNC Symbol;Acc:HGNC:2289] | 2517 | 0.302 | 0.2819 | Yes |
| 112 | HGF | hepatocyte growth factor [Source:HGNC Symbol;Acc:HGNC:4893] | 2547 | 0.297 | 0.2819 | Yes |
| 113 | NUBPL | nucleotide binding protein like [Source:HGNC Symbol;Acc:HGNC:20278] | 2569 | 0.295 | 0.2825 | Yes |
| 114 | TIGAR | TP53 induced glycolysis regulatory phosphatase [Source:HGNC Symbol;Acc:HGNC:1185] | 2580 | 0.293 | 0.2840 | Yes |
| 115 | NDUFB8 | NADH:ubiquinone oxidoreductase subunit B8 [Source:HGNC Symbol;Acc:HGNC:7703] | 2623 | 0.288 | 0.2830 | No |
| 116 | ATP5F1D | ATP synthase F1 subunit delta [Source:HGNC Symbol;Acc:HGNC:837] | 2701 | 0.279 | 0.2790 | No |
| 117 | VDAC2 | voltage dependent anion channel 2 [Source:HGNC Symbol;Acc:HGNC:12672] | 2734 | 0.276 | 0.2786 | No |
| 118 | ACAA2 | acetyl-CoA acyltransferase 2 [Source:HGNC Symbol;Acc:HGNC:83] | 2779 | 0.272 | 0.2773 | No |
| 119 | ATP2A1 | ATPase sarcoplasmic/endoplasmic reticulum Ca2+ transporting 1 [Source:HGNC Symbol;Acc:HGNC:811] | 2781 | 0.272 | 0.2793 | No |
| 120 | TOMM34 | translocase of outer mitochondrial membrane 34 [Source:HGNC Symbol;Acc:HGNC:15746] | 2793 | 0.270 | 0.2806 | No |
| 121 | ULK2 | unc-51 like autophagy activating kinase 2 [Source:HGNC Symbol;Acc:HGNC:13480] | 2796 | 0.270 | 0.2825 | No |
| 122 | TMEM70 | transmembrane protein 70 [Source:HGNC Symbol;Acc:HGNC:26050] | 2813 | 0.269 | 0.2834 | No |
| 123 | LIG3 | DNA ligase 3 [Source:HGNC Symbol;Acc:HGNC:6600] | 2898 | 0.261 | 0.2787 | No |
| 124 | RNF41 | ring finger protein 41 [Source:HGNC Symbol;Acc:HGNC:18401] | 3005 | 0.249 | 0.2722 | No |
| 125 | RAB29 | "RAB29, member RAS oncogene family [Source:HGNC Symbol;Acc:HGNC:9789]" | 3020 | 0.247 | 0.2731 | No |
| 126 | SSBP1 | single stranded DNA binding protein 1 [Source:HGNC Symbol;Acc:HGNC:11317] | 3038 | 0.246 | 0.2736 | No |
| 127 | BIK | BCL2 interacting killer [Source:HGNC Symbol;Acc:HGNC:1051] | 3057 | 0.244 | 0.2741 | No |
| 128 | NDUFS2 | NADH:ubiquinone oxidoreductase core subunit S2 [Source:HGNC Symbol;Acc:HGNC:7708] | 3072 | 0.243 | 0.2749 | No |
| 129 | COX7A2 | cytochrome c oxidase subunit 7A2 [Source:HGNC Symbol;Acc:HGNC:2288] | 3164 | 0.234 | 0.2695 | No |
| 130 | IFIT2 | interferon induced protein with tetratricopeptide repeats 2 [Source:HGNC Symbol;Acc:HGNC:5409] | 3165 | 0.234 | 0.2713 | No |
| 131 | PID1 | phosphotyrosine interaction domain containing 1 [Source:HGNC Symbol;Acc:HGNC:26084] | 3280 | 0.222 | 0.2640 | No |
| 132 | GBA | glucosylceramidase beta [Source:HGNC Symbol;Acc:HGNC:4177] | 3327 | 0.219 | 0.2620 | No |
| 133 | SLIRP | SRA stem-loop interacting RNA binding protein [Source:HGNC Symbol;Acc:HGNC:20495] | 3348 | 0.217 | 0.2621 | No |
| 134 | TIMM22 | translocase of inner mitochondrial membrane 22 [Source:HGNC Symbol;Acc:HGNC:17317] | 3355 | 0.216 | 0.2634 | No |
| 135 | AGK | acylglycerol kinase [Source:HGNC Symbol;Acc:HGNC:21869] | 3373 | 0.214 | 0.2637 | No |
| 136 | CDK5 | cyclin dependent kinase 5 [Source:HGNC Symbol;Acc:HGNC:1774] | 3400 | 0.211 | 0.2633 | No |
| 137 | PARP1 | poly(ADP-ribose) polymerase 1 [Source:HGNC Symbol;Acc:HGNC:270] | 3410 | 0.210 | 0.2642 | No |
| 138 | SAE1 | SUMO1 activating enzyme subunit 1 [Source:HGNC Symbol;Acc:HGNC:30660] | 3423 | 0.209 | 0.2649 | No |
| 139 | TOMM7 | translocase of outer mitochondrial membrane 7 [Source:HGNC Symbol;Acc:HGNC:21648] | 3445 | 0.207 | 0.2649 | No |
| 140 | RAB38 | "RAB38, member RAS oncogene family [Source:HGNC Symbol;Acc:HGNC:9776]" | 3569 | 0.197 | 0.2566 | No |
| 141 | TYMP | thymidine phosphorylase [Source:HGNC Symbol;Acc:HGNC:3148] | 3588 | 0.195 | 0.2567 | No |
| 142 | BAD | BCL2 associated agonist of cell death [Source:HGNC Symbol;Acc:HGNC:936] | 3788 | 0.178 | 0.2422 | No |
| 143 | CDC37 | "cell division cycle 37, HSP90 cochaperone [Source:HGNC Symbol;Acc:HGNC:1735]" | 3810 | 0.175 | 0.2419 | No |
| 144 | IFI6 | interferon alpha inducible protein 6 [Source:HGNC Symbol;Acc:HGNC:4054] | 3812 | 0.175 | 0.2432 | No |
| 145 | NDUFA6 | NADH:ubiquinone oxidoreductase subunit A6 [Source:HGNC Symbol;Acc:HGNC:7690] | 3834 | 0.173 | 0.2429 | No |
| 146 | IMMT | inner membrane mitochondrial protein [Source:HGNC Symbol;Acc:HGNC:6047] | 3864 | 0.168 | 0.2419 | No |
| 147 | VDAC1 | voltage dependent anion channel 1 [Source:HGNC Symbol;Acc:HGNC:12669] | 3939 | 0.161 | 0.2372 | No |
| 148 | MAP1LC3C | microtubule associated protein 1 light chain 3 gamma [Source:HGNC Symbol;Acc:HGNC:13353] | 3943 | 0.161 | 0.2382 | No |
| 149 | TRAK1 | trafficking kinesin protein 1 [Source:HGNC Symbol;Acc:HGNC:29947] | 3964 | 0.159 | 0.2379 | No |
| 150 | MAPT | microtubule associated protein tau [Source:HGNC Symbol;Acc:HGNC:6893] | 3976 | 0.158 | 0.2383 | No |
| 151 | SLC25A46 | solute carrier family 25 member 46 [Source:HGNC Symbol;Acc:HGNC:25198] | 3987 | 0.157 | 0.2387 | No |
| 152 | CIDEB | cell death inducing DFFA like effector b [Source:HGNC Symbol;Acc:HGNC:1977] | 3992 | 0.157 | 0.2396 | No |
| 153 | CNP | "2',3'-cyclic nucleotide 3' phosphodiesterase [Source:HGNC Symbol;Acc:HGNC:2158]" | 4022 | 0.154 | 0.2385 | No |
| 154 | HSD17B10 | hydroxysteroid 17-beta dehydrogenase 10 [Source:HGNC Symbol;Acc:HGNC:4800] | 4060 | 0.150 | 0.2367 | No |
| 155 | ENDOG | endonuclease G [Source:HGNC Symbol;Acc:HGNC:3346] | 4091 | 0.147 | 0.2355 | No |
| 156 | PTCD2 | pentatricopeptide repeat domain 2 [Source:HGNC Symbol;Acc:HGNC:25734] | 4097 | 0.146 | 0.2363 | No |
| 157 | MTCH2 | mitochondrial carrier 2 [Source:HGNC Symbol;Acc:HGNC:17587] | 4147 | 0.142 | 0.2335 | No |
| 158 | SLC35F6 | solute carrier family 35 member F6 [Source:HGNC Symbol;Acc:HGNC:26055] | 4154 | 0.142 | 0.2341 | No |
| 159 | WIPI1 | "WD repeat domain, phosphoinositide interacting 1 [Source:HGNC Symbol;Acc:HGNC:25471]" | 4188 | 0.139 | 0.2326 | No |
| 160 | MAIP1 | matrix AAA peptidase interacting protein 1 [Source:HGNC Symbol;Acc:HGNC:26198] | 4217 | 0.136 | 0.2314 | No |
| 161 | SLC9A1 | solute carrier family 9 member A1 [Source:HGNC Symbol;Acc:HGNC:11071] | 4221 | 0.135 | 0.2322 | No |
| 162 | MIPEP | mitochondrial intermediate peptidase [Source:HGNC Symbol;Acc:HGNC:7104] | 4278 | 0.130 | 0.2288 | No |
| 163 | SDHAF1 | succinate dehydrogenase complex assembly factor 1 [Source:HGNC Symbol;Acc:HGNC:33867] | 4303 | 0.128 | 0.2279 | No |
| 164 | PHB2 | prohibitin 2 [Source:HGNC Symbol;Acc:HGNC:30306] | 4354 | 0.124 | 0.2248 | No |
| 165 | NDUFAF1 | NADH:ubiquinone oxidoreductase complex assembly factor 1 [Source:HGNC Symbol;Acc:HGNC:18828] | 4388 | 0.122 | 0.2232 | No |
| 166 | ATG3 | autophagy related 3 [Source:HGNC Symbol;Acc:HGNC:20962] | 4414 | 0.120 | 0.2221 | No |
| 167 | P2RX7 | purinergic receptor P2X 7 [Source:HGNC Symbol;Acc:HGNC:8537] | 4415 | 0.120 | 0.2231 | No |
| 168 | THG1L | tRNA-histidine guanylyltransferase 1 like [Source:HGNC Symbol;Acc:HGNC:26053] | 4417 | 0.120 | 0.2239 | No |
| 169 | GOLPH3 | golgi phosphoprotein 3 [Source:HGNC Symbol;Acc:HGNC:15452] | 4420 | 0.119 | 0.2247 | No |
| 170 | NDUFAB1 | NADH:ubiquinone oxidoreductase subunit AB1 [Source:HGNC Symbol;Acc:HGNC:7694] | 4445 | 0.118 | 0.2237 | No |
| 171 | FEZ1 | fasciculation and elongation protein zeta 1 [Source:HGNC Symbol;Acc:HGNC:3659] | 4466 | 0.116 | 0.2230 | No |
| 172 | SIRT4 | sirtuin 4 [Source:HGNC Symbol;Acc:HGNC:14932] | 4477 | 0.115 | 0.2231 | No |
| 173 | TOMM70 | translocase of outer mitochondrial membrane 70 [Source:HGNC Symbol;Acc:HGNC:11985] | 4553 | 0.109 | 0.2180 | No |
| 174 | PLSCR3 | phospholipid scramblase 3 [Source:HGNC Symbol;Acc:HGNC:16495] | 4557 | 0.109 | 0.2186 | No |
| 175 | TIMM9 | translocase of inner mitochondrial membrane 9 [Source:HGNC Symbol;Acc:HGNC:11819] | 4603 | 0.104 | 0.2158 | No |
| 176 | EDNRA | endothelin receptor type A [Source:HGNC Symbol;Acc:HGNC:3179] | 4663 | 0.099 | 0.2119 | No |
| 177 | RETREG1 | reticulophagy regulator 1 [Source:HGNC Symbol;Acc:HGNC:25964] | 4674 | 0.098 | 0.2119 | No |
| 178 | SREBF1 | sterol regulatory element binding transcription factor 1 [Source:HGNC Symbol;Acc:HGNC:11289] | 4729 | 0.093 | 0.2083 | No |
| 179 | VAT1 | vesicle amine transport 1 [Source:HGNC Symbol;Acc:HGNC:16919] | 4779 | 0.088 | 0.2051 | No |
| 180 | ATP7A | ATPase copper transporting alpha [Source:HGNC Symbol;Acc:HGNC:869] | 4814 | 0.086 | 0.2030 | No |
| 181 | TIMM17B | translocase of inner mitochondrial membrane 17B [Source:HGNC Symbol;Acc:HGNC:17310] | 4855 | 0.083 | 0.2005 | No |
| 182 | CTSK | cathepsin K [Source:HGNC Symbol;Acc:HGNC:2536] | 4869 | 0.082 | 0.2001 | No |
| 183 | AURKA | aurora kinase A [Source:HGNC Symbol;Acc:HGNC:11393] | 4916 | 0.079 | 0.1970 | No |
| 184 | CAV2 | caveolin 2 [Source:HGNC Symbol;Acc:HGNC:1528] | 4924 | 0.078 | 0.1971 | No |
| 185 | BNIP3L | BCL2 interacting protein 3 like [Source:HGNC Symbol;Acc:HGNC:1085] | 4972 | 0.075 | 0.1939 | No |
| 186 | RAB5IF | RAB5 interacting factor [Source:HGNC Symbol;Acc:HGNC:15870] | 5062 | 0.068 | 0.1874 | No |
| 187 | SLC25A4 | solute carrier family 25 member 4 [Source:HGNC Symbol;Acc:HGNC:10990] | 5073 | 0.067 | 0.1871 | No |
| 188 | PHB | prohibitin [Source:HGNC Symbol;Acc:HGNC:8912] | 5102 | 0.064 | 0.1853 | No |
| 189 | COX7A2P2 | cytochrome c oxidase subunit 7A2 pseudogene 2 [Source:HGNC Symbol;Acc:HGNC:2290] | 5263 | 0.051 | 0.1730 | No |
| 190 | UQCC1 | ubiquinol-cytochrome c reductase complex assembly factor 1 [Source:HGNC Symbol;Acc:HGNC:15891] | 5281 | 0.049 | 0.1720 | No |
| 191 | TIMM23 | translocase of inner mitochondrial membrane 23 [Source:HGNC Symbol;Acc:HGNC:17312] | 5296 | 0.048 | 0.1712 | No |
| 192 | OPTN | optineurin [Source:HGNC Symbol;Acc:HGNC:17142] | 5323 | 0.046 | 0.1695 | No |
| 193 | HIGD1A | HIG1 hypoxia inducible domain family member 1A [Source:HGNC Symbol;Acc:HGNC:29527] | 5345 | 0.044 | 0.1682 | No |
| 194 | TOMM22 | translocase of outer mitochondrial membrane 22 [Source:HGNC Symbol;Acc:HGNC:18002] | 5391 | 0.041 | 0.1649 | No |
| 195 | FZD5 | frizzled class receptor 5 [Source:HGNC Symbol;Acc:HGNC:4043] | 5392 | 0.041 | 0.1652 | No |
| 196 | UBE2L3 | ubiquitin conjugating enzyme E2 L3 [Source:HGNC Symbol;Acc:HGNC:12488] | 5428 | 0.038 | 0.1627 | No |
| 197 | PMPCA | "peptidase, mitochondrial processing subunit alpha [Source:HGNC Symbol;Acc:HGNC:18667]" | 5454 | 0.037 | 0.1610 | No |
| 198 | ATG7 | autophagy related 7 [Source:HGNC Symbol;Acc:HGNC:16935] | 5488 | 0.033 | 0.1587 | No |
| 199 | BNIP3 | BCL2 interacting protein 3 [Source:HGNC Symbol;Acc:HGNC:1084] | 5501 | 0.032 | 0.1580 | No |
| 200 | UQCRFS1 | "ubiquinol-cytochrome c reductase, Rieske iron-sulfur polypeptide 1 [Source:HGNC Symbol;Acc:HGNC:12587]" | 5509 | 0.032 | 0.1576 | No |
| 201 | TP53 | tumor protein p53 [Source:HGNC Symbol;Acc:HGNC:11998] | 5511 | 0.032 | 0.1578 | No |
| 202 | YME1L1 | YME1 like 1 ATPase [Source:HGNC Symbol;Acc:HGNC:12843] | 5528 | 0.030 | 0.1568 | No |
| 203 | TIMM10B | translocase of inner mitochondrial membrane 10B [Source:HGNC Symbol;Acc:HGNC:4022] | 5543 | 0.029 | 0.1559 | No |
| 204 | CLN8 | CLN8 transmembrane ER and ERGIC protein [Source:HGNC Symbol;Acc:HGNC:2079] | 5569 | 0.027 | 0.1541 | No |
| 205 | ATG2B | autophagy related 2B [Source:HGNC Symbol;Acc:HGNC:20187] | 5600 | 0.024 | 0.1519 | No |
| 206 | LEPROT | leptin receptor overlapping transcript [Source:HGNC Symbol;Acc:HGNC:29477] | 5608 | 0.023 | 0.1515 | No |
| 207 | BCL2 | BCL2 apoptosis regulator [Source:HGNC Symbol;Acc:HGNC:990] | 5609 | 0.023 | 0.1517 | No |
| 208 | MEF2A | myocyte enhancer factor 2A [Source:HGNC Symbol;Acc:HGNC:6993] | 5628 | 0.022 | 0.1504 | No |
| 209 | PMPCB | "peptidase, mitochondrial processing subunit beta [Source:HGNC Symbol;Acc:HGNC:9119]" | 5630 | 0.022 | 0.1505 | No |
| 210 | BCL2L2 | BCL2 like 2 [Source:HGNC Symbol;Acc:HGNC:995] | 5643 | 0.020 | 0.1497 | No |
| 211 | TMEM135 | transmembrane protein 135 [Source:HGNC Symbol;Acc:HGNC:26167] | 5646 | 0.020 | 0.1497 | No |
| 212 | FANCG | FA complementation group G [Source:HGNC Symbol;Acc:HGNC:3588] | 5675 | 0.017 | 0.1476 | No |
| 213 | GABARAP | GABA type A receptor-associated protein [Source:HGNC Symbol;Acc:HGNC:4067] | 5707 | 0.015 | 0.1453 | No |
| 214 | BID | BH3 interacting domain death agonist [Source:HGNC Symbol;Acc:HGNC:1050] | 5715 | 0.014 | 0.1448 | No |
| 215 | MTM1 | myotubularin 1 [Source:HGNC Symbol;Acc:HGNC:7448] | 5768 | 0.010 | 0.1407 | No |
| 216 | TERT | telomerase reverse transcriptase [Source:HGNC Symbol;Acc:HGNC:11730] | 5773 | 0.010 | 0.1405 | No |
| 217 | VPS13A | vacuolar protein sorting 13 homolog A [Source:HGNC Symbol;Acc:HGNC:1908] | 5793 | 0.009 | 0.1390 | No |
| 218 | BAG4 | BAG cochaperone 4 [Source:HGNC Symbol;Acc:HGNC:940] | 5857 | 0.005 | 0.1340 | No |
| 219 | POLG2 | "DNA polymerase gamma 2, accessory subunit [Source:HGNC Symbol;Acc:HGNC:9180]" | 5892 | 0.003 | 0.1313 | No |
| 220 | APOOL | apolipoprotein O like [Source:HGNC Symbol;Acc:HGNC:24009] | 5963 | -0.002 | 0.1258 | No |
| 221 | OXA1L | OXA1L mitochondrial inner membrane protein [Source:HGNC Symbol;Acc:HGNC:8526] | 5974 | -0.003 | 0.1250 | No |
| 222 | POLDIP2 | DNA polymerase delta interacting protein 2 [Source:HGNC Symbol;Acc:HGNC:23781] | 5990 | -0.004 | 0.1238 | No |
| 223 | PPARG | peroxisome proliferator activated receptor gamma [Source:HGNC Symbol;Acc:HGNC:9236] | 6006 | -0.005 | 0.1226 | No |
| 224 | NDUFAF4 | NADH:ubiquinone oxidoreductase complex assembly factor 4 [Source:HGNC Symbol;Acc:HGNC:21034] | 6087 | -0.011 | 0.1163 | No |
| 225 | TIMM13 | translocase of inner mitochondrial membrane 13 [Source:HGNC Symbol;Acc:HGNC:11816] | 6090 | -0.012 | 0.1163 | No |
| 226 | COX10 | cytochrome c oxidase assembly factor heme A:farnesyltransferase COX10 [Source:HGNC Symbol;Acc:HGNC:2260] | 6092 | -0.012 | 0.1163 | No |
| 227 | RAC2 | Rac family small GTPase 2 [Source:HGNC Symbol;Acc:HGNC:9802] | 6098 | -0.013 | 0.1160 | No |
| 228 | CSNK2A2 | casein kinase 2 alpha 2 [Source:HGNC Symbol;Acc:HGNC:2459] | 6105 | -0.013 | 0.1156 | No |
| 229 | GZMB | granzyme B [Source:HGNC Symbol;Acc:HGNC:4709] | 6132 | -0.015 | 0.1136 | No |
| 230 | NDUFAF5 | NADH:ubiquinone oxidoreductase complex assembly factor 5 [Source:HGNC Symbol;Acc:HGNC:15899] | 6167 | -0.018 | 0.1111 | No |
| 231 | AMBRA1 | autophagy and beclin 1 regulator 1 [Source:HGNC Symbol;Acc:HGNC:25990] | 6213 | -0.022 | 0.1076 | No |
| 232 | SLC25A36 | solute carrier family 25 member 36 [Source:HGNC Symbol;Acc:HGNC:25554] | 6232 | -0.023 | 0.1064 | No |
| 233 | SQSTM1 | sequestosome 1 [Source:HGNC Symbol;Acc:HGNC:11280] | 6260 | -0.025 | 0.1044 | No |
| 234 | ATP13A2 | ATPase cation transporting 13A2 [Source:HGNC Symbol;Acc:HGNC:30213] | 6336 | -0.031 | 0.0987 | No |
| 235 | TOMM20 | translocase of outer mitochondrial membrane 20 [Source:HGNC Symbol;Acc:HGNC:20947] | 6339 | -0.031 | 0.0988 | No |
| 236 | LMAN1 | "lectin, mannose binding 1 [Source:HGNC Symbol;Acc:HGNC:6631]" | 6361 | -0.032 | 0.0973 | No |
| 237 | PLAUR | "plasminogen activator, urokinase receptor [Source:HGNC Symbol;Acc:HGNC:9053]" | 6362 | -0.032 | 0.0976 | No |
| 238 | RAB3A | "RAB3A, member RAS oncogene family [Source:HGNC Symbol;Acc:HGNC:9777]" | 6440 | -0.038 | 0.0917 | No |
| 239 | DDHD2 | DDHD domain containing 2 [Source:HGNC Symbol;Acc:HGNC:29106] | 6517 | -0.043 | 0.0860 | No |
| 240 | RHOT1 | ras homolog family member T1 [Source:HGNC Symbol;Acc:HGNC:21168] | 6539 | -0.045 | 0.0847 | No |
| 241 | HUWE1 | "HECT, UBA and WWE domain containing E3 ubiquitin protein ligase 1 [Source:HGNC Symbol;Acc:HGNC:30892]" | 6558 | -0.046 | 0.0836 | No |
| 242 | WDR45B | WD repeat domain 45B [Source:HGNC Symbol;Acc:HGNC:25072] | 6560 | -0.047 | 0.0839 | No |
| 243 | SLC4A5 | solute carrier family 4 member 5 [Source:HGNC Symbol;Acc:HGNC:18168] | 6572 | -0.047 | 0.0834 | No |
| 244 | ATG2A | autophagy related 2A [Source:HGNC Symbol;Acc:HGNC:29028] | 6626 | -0.052 | 0.0796 | No |
| 245 | BCL2L1 | BCL2 like 1 [Source:HGNC Symbol;Acc:HGNC:992] | 6698 | -0.055 | 0.0743 | No |
| 246 | NDUFA5 | NADH:ubiquinone oxidoreductase subunit A5 [Source:HGNC Symbol;Acc:HGNC:7688] | 6789 | -0.062 | 0.0676 | No |
| 247 | FZD9 | frizzled class receptor 9 [Source:HGNC Symbol;Acc:HGNC:4047] | 6812 | -0.064 | 0.0663 | No |
| 248 | ERBB4 | erb-b2 receptor tyrosine kinase 4 [Source:HGNC Symbol;Acc:HGNC:3432] | 6830 | -0.065 | 0.0655 | No |
| 249 | MTFR1 | mitochondrial fission regulator 1 [Source:HGNC Symbol;Acc:HGNC:29510] | 6890 | -0.071 | 0.0613 | No |
| 250 | TSC2 | TSC complex subunit 2 [Source:HGNC Symbol;Acc:HGNC:12363] | 6946 | -0.075 | 0.0575 | No |
| 251 | MTERF4 | mitochondrial transcription termination factor 4 [Source:HGNC Symbol;Acc:HGNC:28785] | 6972 | -0.078 | 0.0562 | No |
| 252 | RB1CC1 | RB1 inducible coiled-coil 1 [Source:HGNC Symbol;Acc:HGNC:15574] | 7056 | -0.084 | 0.0502 | No |
| 253 | MFN2 | mitofusin 2 [Source:HGNC Symbol;Acc:HGNC:16877] | 7064 | -0.085 | 0.0503 | No |
| 254 | SHARPIN | SHANK associated RH domain interactor [Source:HGNC Symbol;Acc:HGNC:25321] | 7119 | -0.090 | 0.0467 | No |
| 255 | MLLT11 | MLLT11 transcription factor 7 cofactor [Source:HGNC Symbol;Acc:HGNC:16997] | 7130 | -0.091 | 0.0466 | No |
| 256 | TIMM8A | translocase of inner mitochondrial membrane 8A [Source:HGNC Symbol;Acc:HGNC:11817] | 7138 | -0.092 | 0.0468 | No |
| 257 | GDAP1 | ganglioside induced differentiation associated protein 1 [Source:HGNC Symbol;Acc:HGNC:15968] | 7172 | -0.093 | 0.0449 | No |
| 258 | AVP | arginine vasopressin [Source:HGNC Symbol;Acc:HGNC:894] | 7201 | -0.096 | 0.0434 | No |
| 259 | ARIH2 | ariadne RBR E3 ubiquitin protein ligase 2 [Source:HGNC Symbol;Acc:HGNC:690] | 7226 | -0.097 | 0.0422 | No |
| 260 | HEBP2 | heme binding protein 2 [Source:HGNC Symbol;Acc:HGNC:15716] | 7252 | -0.099 | 0.0410 | No |
| 261 | ARRB2 | arrestin beta 2 [Source:HGNC Symbol;Acc:HGNC:712] | 7255 | -0.100 | 0.0416 | No |
| 262 | PPP2CB | protein phosphatase 2 catalytic subunit beta [Source:HGNC Symbol;Acc:HGNC:9300] | 7265 | -0.100 | 0.0417 | No |
| 263 | VPS13D | vacuolar protein sorting 13 homolog D [Source:HGNC Symbol;Acc:HGNC:23595] | 7307 | -0.103 | 0.0393 | No |
| 264 | MAP1LC3B | microtubule associated protein 1 light chain 3 beta [Source:HGNC Symbol;Acc:HGNC:13352] | 7323 | -0.104 | 0.0389 | No |
| 265 | ATG9A | autophagy related 9A [Source:HGNC Symbol;Acc:HGNC:22408] | 7391 | -0.110 | 0.0344 | No |
| 266 | COA4 | cytochrome c oxidase assembly factor 4 homolog [Source:HGNC Symbol;Acc:HGNC:24604] | 7460 | -0.115 | 0.0298 | No |
| 267 | LONP1 | "lon peptidase 1, mitochondrial [Source:HGNC Symbol;Acc:HGNC:9479]" | 7531 | -0.121 | 0.0252 | No |
| 268 | NOL3 | nucleolar protein 3 [Source:HGNC Symbol;Acc:HGNC:7869] | 7545 | -0.122 | 0.0251 | No |
| 269 | BCS1L | "BCS1 homolog, ubiquinol-cytochrome c reductase complex chaperone [Source:HGNC Symbol;Acc:HGNC:1020]" | 7578 | -0.125 | 0.0236 | No |
| 270 | HTT | huntingtin [Source:HGNC Symbol;Acc:HGNC:4851] | 7641 | -0.130 | 0.0196 | No |
| 271 | NMT1 | N-myristoyltransferase 1 [Source:HGNC Symbol;Acc:HGNC:7857] | 7758 | -0.138 | 0.0114 | No |
| 272 | HDAC6 | histone deacetylase 6 [Source:HGNC Symbol;Acc:HGNC:14064] | 7833 | -0.143 | 0.0067 | No |
| 273 | HPS4 | HPS4 biogenesis of lysosomal organelles complex 3 subunit 2 [Source:HGNC Symbol;Acc:HGNC:15844] | 7868 | -0.146 | 0.0051 | No |
| 274 | HSPA1A | heat shock protein family A (Hsp70) member 1A [Source:HGNC Symbol;Acc:HGNC:5232] | 7876 | -0.146 | 0.0057 | No |
| 275 | SLC25A5 | solute carrier family 25 member 5 [Source:HGNC Symbol;Acc:HGNC:10991] | 7877 | -0.146 | 0.0068 | No |
| 276 | IAPP | islet amyloid polypeptide [Source:HGNC Symbol;Acc:HGNC:5329] | 7976 | -0.153 | 0.0002 | No |
| 277 | AKT3 | AKT serine/threonine kinase 3 [Source:HGNC Symbol;Acc:HGNC:393] | 8008 | -0.156 | -0.0010 | No |
| 278 | MYH14 | myosin heavy chain 14 [Source:HGNC Symbol;Acc:HGNC:23212] | 8032 | -0.158 | -0.0016 | No |
| 279 | FXN | frataxin [Source:HGNC Symbol;Acc:HGNC:3951] | 8057 | -0.160 | -0.0023 | No |
| 280 | TIMM17A | translocase of inner mitochondrial membrane 17A [Source:HGNC Symbol;Acc:HGNC:17315] | 8076 | -0.161 | -0.0025 | No |
| 281 | SFN | stratifin [Source:HGNC Symbol;Acc:HGNC:10773] | 8118 | -0.164 | -0.0044 | No |
| 282 | DMAC2 | distal membrane arm assembly component 2 [Source:HGNC Symbol;Acc:HGNC:25496] | 8347 | -0.182 | -0.0212 | No |
| 283 | MFN1 | mitofusin 1 [Source:HGNC Symbol;Acc:HGNC:18262] | 8460 | -0.191 | -0.0287 | No |
| 284 | ZNF205 | zinc finger protein 205 [Source:HGNC Symbol;Acc:HGNC:12996] | 8465 | -0.191 | -0.0275 | No |
| 285 | PITRM1 | pitrilysin metallopeptidase 1 [Source:HGNC Symbol;Acc:HGNC:17663] | 8476 | -0.192 | -0.0268 | No |
| 286 | CAMKMT | calmodulin-lysine N-methyltransferase [Source:HGNC Symbol;Acc:HGNC:26276] | 8484 | -0.193 | -0.0258 | No |
| 287 | DNM1L | dynamin 1 like [Source:HGNC Symbol;Acc:HGNC:2973] | 8499 | -0.194 | -0.0254 | No |
| 288 | TMEM11 | transmembrane protein 11 [Source:HGNC Symbol;Acc:HGNC:16823] | 8520 | -0.195 | -0.0255 | No |
| 289 | GCLC | glutamate-cysteine ligase catalytic subunit [Source:HGNC Symbol;Acc:HGNC:4311] | 8534 | -0.196 | -0.0250 | No |
| 290 | ARMCX3 | armadillo repeat containing X-linked 3 [Source:HGNC Symbol;Acc:HGNC:24065] | 8560 | -0.198 | -0.0254 | No |
| 291 | HIGD1B | HIG1 hypoxia inducible domain family member 1B [Source:HGNC Symbol;Acc:HGNC:24318] | 8575 | -0.200 | -0.0249 | No |
| 292 | ATF2 | activating transcription factor 2 [Source:HGNC Symbol;Acc:HGNC:784] | 8592 | -0.201 | -0.0246 | No |
| 293 | BECN1 | beclin 1 [Source:HGNC Symbol;Acc:HGNC:1034] | 8644 | -0.204 | -0.0271 | No |
| 294 | OPA1 | OPA1 mitochondrial dynamin like GTPase [Source:HGNC Symbol;Acc:HGNC:8140] | 8655 | -0.205 | -0.0263 | No |
| 295 | MMP9 | matrix metallopeptidase 9 [Source:HGNC Symbol;Acc:HGNC:7176] | 8698 | -0.209 | -0.0280 | No |
| 296 | TIMM50 | translocase of inner mitochondrial membrane 50 [Source:HGNC Symbol;Acc:HGNC:23656] | 8731 | -0.211 | -0.0289 | No |
| 297 | SIRT7 | sirtuin 7 [Source:HGNC Symbol;Acc:HGNC:14935] | 8733 | -0.211 | -0.0273 | No |
| 298 | GABARAPL3 | GABA type A receptor associated protein like 3 pseudogene [Source:HGNC Symbol;Acc:HGNC:4069] | 8739 | -0.212 | -0.0260 | No |
| 299 | CNTNAP1 | contactin associated protein 1 [Source:HGNC Symbol;Acc:HGNC:8011] | 8809 | -0.218 | -0.0298 | No |
| 300 | FLCN | folliculin [Source:HGNC Symbol;Acc:HGNC:27310] | 8848 | -0.221 | -0.0311 | No |
| 301 | PDE2A | phosphodiesterase 2A [Source:HGNC Symbol;Acc:HGNC:8777] | 8872 | -0.223 | -0.0312 | No |
| 302 | GABPB1 | GA binding protein transcription factor subunit beta 1 [Source:HGNC Symbol;Acc:HGNC:4074] | 8900 | -0.225 | -0.0316 | No |
| 303 | MAPK8 | mitogen-activated protein kinase 8 [Source:HGNC Symbol;Acc:HGNC:6881] | 8956 | -0.228 | -0.0342 | No |
| 304 | ARFIP2 | ADP ribosylation factor interacting protein 2 [Source:HGNC Symbol;Acc:HGNC:17160] | 8976 | -0.230 | -0.0339 | No |
| 305 | GRPEL1 | "GrpE like 1, mitochondrial [Source:HGNC Symbol;Acc:HGNC:19696]" | 9026 | -0.234 | -0.0360 | No |
| 306 | PDCD5 | programmed cell death 5 [Source:HGNC Symbol;Acc:HGNC:8764] | 9039 | -0.235 | -0.0351 | No |
| 307 | AFG3L2 | AFG3 like matrix AAA peptidase subunit 2 [Source:HGNC Symbol;Acc:HGNC:315] | 9045 | -0.235 | -0.0336 | No |
| 308 | DNAJA3 | DnaJ heat shock protein family (Hsp40) member A3 [Source:HGNC Symbol;Acc:HGNC:11808] | 9172 | -0.246 | -0.0418 | No |
| 309 | HSPA9 | heat shock protein family A (Hsp70) member 9 [Source:HGNC Symbol;Acc:HGNC:5244] | 9173 | -0.246 | -0.0398 | No |
| 310 | BAK1 | BCL2 antagonist/killer 1 [Source:HGNC Symbol;Acc:HGNC:949] | 9266 | -0.253 | -0.0452 | No |
| 311 | BAX | "BCL2 associated X, apoptosis regulator [Source:HGNC Symbol;Acc:HGNC:959]" | 9323 | -0.257 | -0.0476 | No |
| 312 | TRAK2 | trafficking kinesin protein 2 [Source:HGNC Symbol;Acc:HGNC:13206] | 9411 | -0.265 | -0.0525 | No |
| 313 | RHOT2 | ras homolog family member T2 [Source:HGNC Symbol;Acc:HGNC:21169] | 9476 | -0.272 | -0.0555 | No |
| 314 | ATG13 | autophagy related 13 [Source:HGNC Symbol;Acc:HGNC:29091] | 9644 | -0.287 | -0.0666 | No |
| 315 | MARK2 | microtubule affinity regulating kinase 2 [Source:HGNC Symbol;Acc:HGNC:3332] | 9662 | -0.288 | -0.0656 | No |
| 316 | ATG12 | autophagy related 12 [Source:HGNC Symbol;Acc:HGNC:588] | 9776 | -0.299 | -0.0723 | No |
| 317 | HSPA4 | heat shock protein family A (Hsp70) member 4 [Source:HGNC Symbol;Acc:HGNC:5237] | 9797 | -0.301 | -0.0715 | No |
| 318 | ULK1 | unc-51 like autophagy activating kinase 1 [Source:HGNC Symbol;Acc:HGNC:12558] | 9798 | -0.301 | -0.0692 | No |
| 319 | KAT2A | lysine acetyltransferase 2A [Source:HGNC Symbol;Acc:HGNC:4201] | 9799 | -0.301 | -0.0668 | No |
| 320 | NOS3 | nitric oxide synthase 3 [Source:HGNC Symbol;Acc:HGNC:7876] | 9980 | -0.319 | -0.0787 | No |
| 321 | PPIF | peptidylprolyl isomerase F [Source:HGNC Symbol;Acc:HGNC:9259] | 9999 | -0.321 | -0.0776 | No |
| 322 | HK2 | hexokinase 2 [Source:HGNC Symbol;Acc:HGNC:4923] | 10030 | -0.325 | -0.0774 | No |
| 323 | INF2 | inverted formin 2 [Source:HGNC Symbol;Acc:HGNC:23791] | 10051 | -0.326 | -0.0764 | No |
| 324 | SREBF2 | sterol regulatory element binding transcription factor 2 [Source:HGNC Symbol;Acc:HGNC:11290] | 10070 | -0.328 | -0.0753 | No |
| 325 | NDUFAF7 | NADH:ubiquinone oxidoreductase complex assembly factor 7 [Source:HGNC Symbol;Acc:HGNC:28816] | 10077 | -0.328 | -0.0732 | No |
| 326 | PMAIP1 | phorbol-12-myristate-13-acetate-induced protein 1 [Source:HGNC Symbol;Acc:HGNC:9108] | 10084 | -0.329 | -0.0711 | No |
| 327 | BAP1 | BRCA1 associated protein 1 [Source:HGNC Symbol;Acc:HGNC:950] | 10151 | -0.336 | -0.0737 | No |
| 328 | NPEPPS | aminopeptidase puromycin sensitive [Source:HGNC Symbol;Acc:HGNC:7900] | 10157 | -0.337 | -0.0714 | No |
| 329 | TFRC | transferrin receptor [Source:HGNC Symbol;Acc:HGNC:11763] | 10184 | -0.339 | -0.0708 | No |
| 330 | NECTIN2 | nectin cell adhesion molecule 2 [Source:HGNC Symbol;Acc:HGNC:9707] | 10204 | -0.341 | -0.0697 | No |
| 331 | ATG4B | autophagy related 4B cysteine peptidase [Source:HGNC Symbol;Acc:HGNC:20790] | 10243 | -0.346 | -0.0700 | No |
| 332 | HRK | "harakiri, BCL2 interacting protein [Source:HGNC Symbol;Acc:HGNC:5185]" | 10383 | -0.359 | -0.0783 | No |
| 333 | RTL10 | retrotransposon Gag like 10 [Source:HGNC Symbol;Acc:HGNC:26112] | 10393 | -0.360 | -0.0761 | No |
| 334 | TAFAZZIN | "tafazzin, phospholipid-lysophospholipid transacylase [Source:HGNC Symbol;Acc:HGNC:11577]" | 10429 | -0.364 | -0.0761 | No |
| 335 | GCLM | glutamate-cysteine ligase modifier subunit [Source:HGNC Symbol;Acc:HGNC:4312] | 10439 | -0.366 | -0.0739 | No |
| 336 | ATG14 | autophagy related 14 [Source:HGNC Symbol;Acc:HGNC:19962] | 10465 | -0.369 | -0.0730 | No |
| 337 | PPARGC1A | PPARG coactivator 1 alpha [Source:HGNC Symbol;Acc:HGNC:9237] | 10504 | -0.374 | -0.0731 | No |
| 338 | GGCT | gamma-glutamylcyclotransferase [Source:HGNC Symbol;Acc:HGNC:21705] | 10515 | -0.375 | -0.0709 | No |
| 339 | ATPAF2 | ATP synthase mitochondrial F1 complex assembly factor 2 [Source:HGNC Symbol;Acc:HGNC:18802] | 10610 | -0.384 | -0.0754 | No |
| 340 | CYRIB | CYFIP related Rac1 interactor B [Source:HGNC Symbol;Acc:HGNC:25216] | 10612 | -0.384 | -0.0724 | No |
| 341 | DNAJC11 | DnaJ heat shock protein family (Hsp40) member C11 [Source:HGNC Symbol;Acc:HGNC:25570] | 10625 | -0.385 | -0.0704 | No |
| 342 | PEX5 | peroxisomal biogenesis factor 5 [Source:HGNC Symbol;Acc:HGNC:9719] | 10737 | -0.398 | -0.0761 | No |
| 343 | AGTPBP1 | ATP/GTP binding carboxypeptidase 1 [Source:HGNC Symbol;Acc:HGNC:17258] | 10752 | -0.399 | -0.0741 | No |
| 344 | PLEC | plectin [Source:HGNC Symbol;Acc:HGNC:9069] | 10799 | -0.405 | -0.0745 | No |
| 345 | MYO19 | myosin XIX [Source:HGNC Symbol;Acc:HGNC:26234] | 10938 | -0.423 | -0.0822 | No |
| 346 | LMNA | lamin A/C [Source:HGNC Symbol;Acc:HGNC:6636] | 10946 | -0.424 | -0.0795 | No |
| 347 | UBE2D3 | ubiquitin conjugating enzyme E2 D3 [Source:HGNC Symbol;Acc:HGNC:12476] | 10967 | -0.427 | -0.0777 | No |
| 348 | MICALL2 | MICAL like 2 [Source:HGNC Symbol;Acc:HGNC:29672] | 11037 | -0.435 | -0.0798 | No |
| 349 | PAM16 | presequence translocase associated motor 16 [Source:HGNC Symbol;Acc:HGNC:29679] | 11121 | -0.446 | -0.0829 | No |
| 350 | PLA2G6 | phospholipase A2 group VI [Source:HGNC Symbol;Acc:HGNC:9039] | 11128 | -0.446 | -0.0799 | No |
| 351 | GSK3B | glycogen synthase kinase 3 beta [Source:HGNC Symbol;Acc:HGNC:4617] | 11157 | -0.451 | -0.0785 | No |
| 352 | EDN1 | endothelin 1 [Source:HGNC Symbol;Acc:HGNC:3176] | 11276 | -0.468 | -0.0843 | No |
| 353 | WASF1 | WASP family member 1 [Source:HGNC Symbol;Acc:HGNC:12732] | 11387 | -0.484 | -0.0893 | No |
| 354 | ABLIM3 | actin binding LIM protein family member 3 [Source:HGNC Symbol;Acc:HGNC:29132] | 11398 | -0.487 | -0.0862 | No |
| 355 | DCN | decorin [Source:HGNC Symbol;Acc:HGNC:2705] | 11402 | -0.487 | -0.0826 | No |
| 356 | SCO2 | synthesis of cytochrome C oxidase 2 [Source:HGNC Symbol;Acc:HGNC:10604] | 11410 | -0.488 | -0.0793 | No |
| 357 | HSPD1 | heat shock protein family D (Hsp60) member 1 [Source:HGNC Symbol;Acc:HGNC:5261] | 11427 | -0.492 | -0.0767 | No |
| 358 | KDR | kinase insert domain receptor [Source:HGNC Symbol;Acc:HGNC:6307] | 11454 | -0.496 | -0.0749 | No |
| 359 | PRKAA1 | protein kinase AMP-activated catalytic subunit alpha 1 [Source:HGNC Symbol;Acc:HGNC:9376] | 11499 | -0.501 | -0.0744 | No |
| 360 | FBXW7 | F-box and WD repeat domain containing 7 [Source:HGNC Symbol;Acc:HGNC:16712] | 11524 | -0.505 | -0.0724 | No |
| 361 | USP36 | ubiquitin specific peptidase 36 [Source:HGNC Symbol;Acc:HGNC:20062] | 11560 | -0.508 | -0.0712 | No |
| 362 | BAG3 | BAG cochaperone 3 [Source:HGNC Symbol;Acc:HGNC:939] | 11562 | -0.509 | -0.0672 | No |
| 363 | CTTN | cortactin [Source:HGNC Symbol;Acc:HGNC:3338] | 11677 | -0.528 | -0.0722 | No |
| 364 | HIP1R | huntingtin interacting protein 1 related [Source:HGNC Symbol;Acc:HGNC:18415] | 11717 | -0.535 | -0.0711 | No |
| 365 | CIBAR1 | CBY1 interacting BAR domain containing 1 [Source:HGNC Symbol;Acc:HGNC:30452] | 11718 | -0.536 | -0.0668 | No |
| 366 | NPTX1 | neuronal pentraxin 1 [Source:HGNC Symbol;Acc:HGNC:7952] | 11733 | -0.539 | -0.0637 | No |
| 367 | TIMM44 | translocase of inner mitochondrial membrane 44 [Source:HGNC Symbol;Acc:HGNC:17316] | 11738 | -0.539 | -0.0598 | No |
| 368 | EP300 | E1A binding protein p300 [Source:HGNC Symbol;Acc:HGNC:3373] | 11741 | -0.540 | -0.0557 | No |
| 369 | EPAS1 | endothelial PAS domain protein 1 [Source:HGNC Symbol;Acc:HGNC:3374] | 11758 | -0.542 | -0.0527 | No |
| 370 | HIF1A | hypoxia inducible factor 1 subunit alpha [Source:HGNC Symbol;Acc:HGNC:4910] | 11833 | -0.556 | -0.0542 | No |
| 371 | BCL2L11 | BCL2 like 11 [Source:HGNC Symbol;Acc:HGNC:994] | 11867 | -0.564 | -0.0524 | No |
| 372 | ATAD3A | ATPase family AAA domain containing 3A [Source:HGNC Symbol;Acc:HGNC:25567] | 11920 | -0.577 | -0.0520 | No |
| 373 | TOMM40 | translocase of outer mitochondrial membrane 40 [Source:HGNC Symbol;Acc:HGNC:18001] | 11977 | -0.589 | -0.0518 | No |
| 374 | SPG7 | "SPG7 matrix AAA peptidase subunit, paraplegin [Source:HGNC Symbol;Acc:HGNC:11237]" | 12020 | -0.597 | -0.0505 | No |
| 375 | SUPV3L1 | Suv3 like RNA helicase [Source:HGNC Symbol;Acc:HGNC:11471] | 12087 | -0.616 | -0.0509 | No |
| 376 | MSTO1 | misato mitochondrial distribution and morphology regulator 1 [Source:HGNC Symbol;Acc:HGNC:29678] | 12113 | -0.623 | -0.0480 | No |
| 377 | DNA2 | DNA replication helicase/nuclease 2 [Source:HGNC Symbol;Acc:HGNC:2939] | 12148 | -0.631 | -0.0457 | No |
| 378 | TFAM | "transcription factor A, mitochondrial [Source:HGNC Symbol;Acc:HGNC:11741]" | 12175 | -0.637 | -0.0428 | No |
| 379 | STAT2 | signal transducer and activator of transcription 2 [Source:HGNC Symbol;Acc:HGNC:11363] | 12196 | -0.644 | -0.0393 | No |
| 380 | LETM1 | leucine zipper and EF-hand containing transmembrane protein 1 [Source:HGNC Symbol;Acc:HGNC:6556] | 12272 | -0.669 | -0.0400 | No |
| 381 | BBC3 | BCL2 binding component 3 [Source:HGNC Symbol;Acc:HGNC:17868] | 12298 | -0.676 | -0.0367 | No |
| 382 | RNF31 | ring finger protein 31 [Source:HGNC Symbol;Acc:HGNC:16031] | 12332 | -0.686 | -0.0339 | No |
| 383 | VPS13C | vacuolar protein sorting 13 homolog C [Source:HGNC Symbol;Acc:HGNC:23594] | 12367 | -0.696 | -0.0311 | No |
| 384 | GSK3A | glycogen synthase kinase 3 alpha [Source:HGNC Symbol;Acc:HGNC:4616] | 12384 | -0.703 | -0.0269 | No |
| 385 | RALA | RAS like proto-oncogene A [Source:HGNC Symbol;Acc:HGNC:9839] | 12398 | -0.707 | -0.0223 | No |
| 386 | CERT1 | ceramide transporter 1 [Source:HGNC Symbol;Acc:HGNC:2205] | 12463 | -0.732 | -0.0217 | No |
| 387 | PISD | phosphatidylserine decarboxylase [Source:HGNC Symbol;Acc:HGNC:8999] | 12581 | -0.785 | -0.0248 | No |
| 388 | GABARAPL1 | GABA type A receptor associated protein like 1 [Source:HGNC Symbol;Acc:HGNC:4068] | 12616 | -0.808 | -0.0212 | No |
| 389 | SOD2 | superoxide dismutase 2 [Source:HGNC Symbol;Acc:HGNC:11180] | 12685 | -0.853 | -0.0199 | No |
| 390 | TOP3A | DNA topoisomerase III alpha [Source:HGNC Symbol;Acc:HGNC:11992] | 12707 | -0.867 | -0.0147 | No |
| 391 | HSP90AA1 | heat shock protein 90 alpha family class A member 1 [Source:HGNC Symbol;Acc:HGNC:5253] | 12730 | -0.893 | -0.0094 | No |
| 392 | MT-ND5 | mitochondrially encoded NADH:ubiquinone oxidoreductase core subunit 5 [Source:HGNC Symbol;Acc:HGNC:7461] | 12743 | -0.906 | -0.0032 | No |
| 393 | MYC | "MYC proto-oncogene, bHLH transcription factor [Source:HGNC Symbol;Acc:HGNC:7553]" | 12801 | -0.970 | -0.0002 | No |
| 394 | CLUH | clustered mitochondria homolog [Source:HGNC Symbol;Acc:HGNC:29094] | 12824 | -1.013 | 0.0061 | No |
Table: GSEA details [plain text format]

  

Fig 2: GOBP\_MITOCHONDRION\_ORGANIZATION      
 Blue-Pink O' Gram in the Space of the Analyzed GeneSet

  

Fig 3: GOBP\_MITOCHONDRION\_ORGANIZATION: Random ES distribution      
 Gene set null distribution of ES for **GOBP\_MITOCHONDRION\_ORGANIZATION**
